# Analysis of Geometric Performance and Dosimetric Impact of Using Automatic Contour Segmentation for Radiotherapy Planning

**DOI:** 10.3389/fonc.2020.01762

**Published:** 2020-09-23

**Authors:** Minsong Cao, Bradley Stiehl, Victoria Y. Yu, Ke Sheng, Amar U. Kishan, Robert K. Chin, Yingli Yang, Dan Ruan

**Affiliations:** ^1^Department of Radiation Oncology, David Geffen School of Medicine, University of California, Los Angeles, Los Angeles, CA, United States; ^2^Physics & Biology in Medicine Graduate Program, University of California, Los Angeles, Los Angeles, CA, United States; ^3^Department of Medical Physics, Memorial Sloan Kettering Cancer Center, New York, NY, United States

**Keywords:** autosegmentation, radiotherapy planning, dosimetry, geometric metrics, contour

## Abstract

**Purpose:** To analyze geometric discrepancy and dosimetric impact in using contours generated by auto-segmentation (AS) against manually segmented (MS) clinical contours.

**Methods:** A 48-subject prostate atlas was created and another 15 patients were used for testing. Contours were generated using a commercial atlas-based segmentation tool and compared to their clinical MS counterparts. The geometric correlation was evaluated using the Dice similarity coefficient (DSC) and Hausdorff distance (HD). Dosimetric relevance was evaluated for a subset of patients by assessing the DVH differences derived by optimizing plan dose using the AS and MS contours, respectively, and evaluating with respect to each. A paired *t*-test was employed for statistical comparison. The discrepancy in plan quality with respect to clinical dosimetric endpoints was evaluated. The analysis was repeated for head/neck (HN) with a 31-subject atlas and 15 test cases.

**Results:** Dice agreement between AS and MS differed significantly across structures: from (L:0.92/R: 0.91) for the femoral heads to seminal vesical of 0.38 in the prostate cohort, and from 0.98 for the brain, to 0.36 for the chiasm of the HN group. Despite the geometric disagreement, the paired *t*-tests showed the lack of statistical evidence for systematic differences in dosimetric plan quality yielded by the AS and MS approach for the prostate cohort. In HN cases, statistically significant differences in dosimetric endpoints were observed in structures with small volumes or elongated shapes such as cord (*p* = 0.01) and esophagus (*p* = 0.04). The largest absolute dose difference of 11 Gy was seen in the mean pharynx dose.

**Conclusion:** Varying AS performance among structures suggests a differential approach of using AS on a subset of structures and focus MS on the rest. The discrepancy between geometric and dosimetric-end-point driven evaluation also indicates the clinical utility of AS contours in optimization and evaluating plan quality despite of suboptimal geometrical accuracy.

## Introduction

Accurate and efficient contouring is essential to the quality of treatment planning in radiation therapy because incorrect delineation of the target volume and organs at risk (OARs) can lead to insufficient target coverage or normal tissue sparing and severe side effects. Conventionally, tumor volumes, and OARs are contoured manually by trained medical professionals. This process is often labor-intensive and subject to inter-/intra-operator variations, which may hinder the efficiency and effectiveness of the clinical operation. In recent years, automatic segmentation (AS) has gained popularity as an alternative or auxiliary method to manual segmentation (MS) (1, 2). Studies have shown that automatic segmentation is capable of significantly reducing the amount of time spent performing this task while producing reasonably similar contours for various treatment sites (3–6). A variety of automated segmentation approaches have been introduced and demonstrated with promising results, including atlas-based segmentation (7–10), statistical models of shape and appearance (11, 12), machine learning-based methods and hybrid approaches (13–16).

One of the major challenges of implementation of automated contour segmentation in clinical practice is the lack of effective validation and evaluation of its accuracy and reliability. Existing literature regarding the evaluation of automatic segmentation primarily considers the geometric agreement between contours created by automatic segmentation and those produced through manual delineation. Common geometric metrics, including moment-based methods, overlap metrics, and distance-based measures (1, 5, 17) have been widely reported in literature and segmentation grand challenges to evaluate the geometric accuracy of the segmented contours (10, 14, 18).

Related but different from the geometrical accuracy measurement of the segmented contours, dosimetric accuracy directly influences the treatment plan quality, and associated clinical decision-making processes. Treatment plans containing contouring deviation in the range of a few millimeters may still have similar dose distribution (1). The impact of the geometric agreement into the dose domain and plan quality remains elusive. This study aims to bridge this gap by investigating the effect of an atlas-based automatic segmentation method on dose optimization and plan evaluation using dosimetric quality metrics. We aim to test the hypothesis that automatic segmentation is capable of providing contours that may be used to generate clinically feasible plans according to established dosimetric plan quality endpoints, despite their geometric inaccuracy.

## Materials and Methods

**Figure 1:**
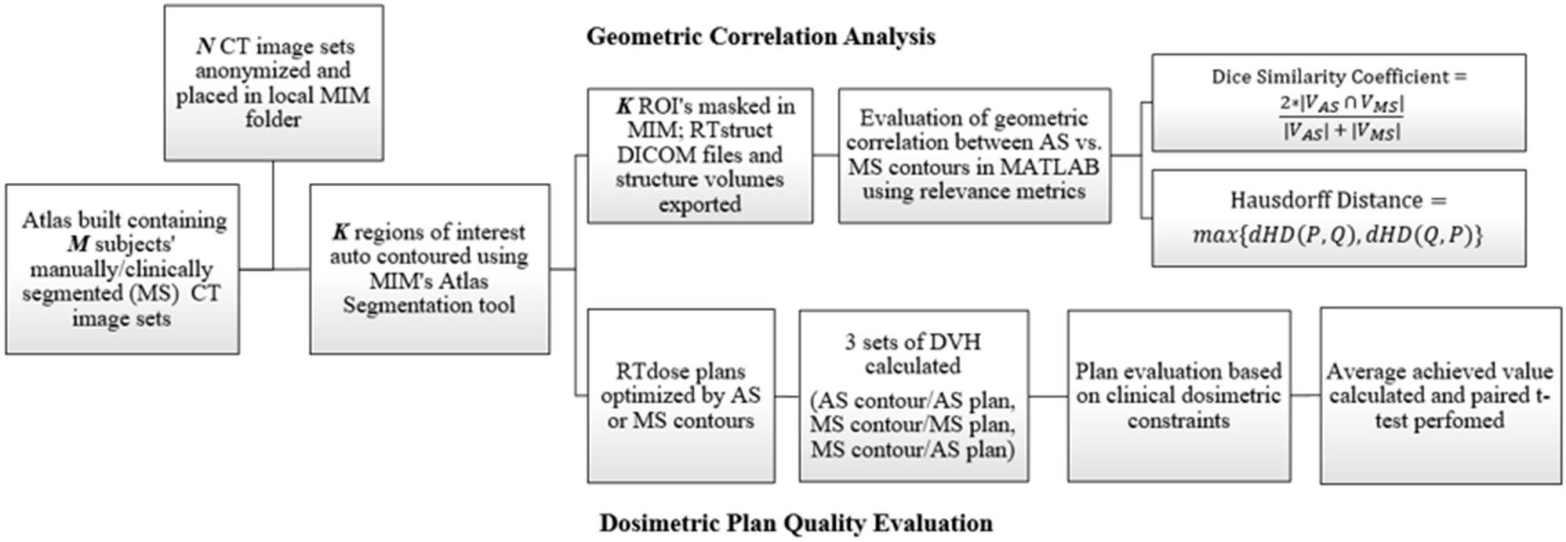
Flowchart showing the methodology for this study which consists of both geometric and dosimetric evaluation.

The methodology and analysis workflow of this study is demonstrated in Figure 1. It consists of four major components, atlas construction, structure auto-segmentation, geometric correlation analysis, and dosimetric plan quality evaluation, which are described in detail in the following sections.

### Data Acquisition and Atlas Construction

This retrospective study was reviewed and approved by Institutional Review Board (IRB) and written informed consent for participation was waived for this study. Patient CT images and associated treatment planning data (manually segmented contours and dose matrix) were de-identified and exported into a commercially available contour toolkit (MIM Software, Cleveland, OH). Atlases of 48 and 31 patients were constructed for the prostate and head and neck (HN) sites, respectively. For both sites, 15 additional subjects were randomly selected and used for testing. The structure set for the prostate consisted of the clinical target volume (CTV) and 6 OARs, including bladder, femoral heads, penile bulb, rectum, and seminal vesicle. For HN, the structure set included 15 OARs (i.e., brain, brainstem, chiasm, spinal cord, esophagus, larynx, lips, mandible, optic nerves, orbits, parotids, and pharynx). Target contours of the HN cases were not included and evaluated in this study due to their complexity and large variations.

### Atlas-Based Segmentation

These respective sets of structures for each site were contoured using the atlas segmentation feature provided in MIM. This process involved the selection of a single best-matched atlas subject, from the atlas built-in section data acquisition and atlas construction, followed by deformable image registration (DIR) of the atlas subject CT to the patient CT (19, 20). The derived deformation vector field subsequently propagated contours on the atlas subject to the target CT images.

### Geometric Correlation

Geometric correlation analysis was performed using an in-house MATLAB tool. Specifically, the Dice Similarity Coefficient (DSC) was used to measure the geometric overlap between the structure regions defined by the AS and MS contour as follows:

(1) $$\begin{array}{l} \text{DSC}=\frac{2|{\text{V}}_{\text{AS }}\cap \;{\text{V}}_{\text{MS}}|}{\left|{\text{V}}_{\text{AS}}|\;+\;|{\text{V}}_{\text{MS}}\right|} \end{array}$$

In addition, the Hausdorff distance (HD) was used to evaluate the boundary accordance between AS and MS contours by the in-house tool where the largest distance between paired points in these two contour sets is calculated (21). A symmetric version in equation (2) is used, based on directional setup:

(2) $$\begin{array}{l} \text{HD }=\text{max }\left\{\text{ dHD}\left(\text{A},\text{ B}\right),\text{ dHD}\left(\text{B},\text{ A}\right)\;\right\} \end{array}$$

Where *directional Hausdorff Distance (dHD)* is:

(3) $$\begin{array}{l} \text{dHD}\left(\text{A},\text{ B}\right)\;=\text{max }\left(\text{ a ϵ A }\right)[\text{min}\text{ b ϵ B }\left[\;||\text{a}-\text{b}||\;\right] \end{array}$$

(4) $$\begin{array}{l} \text{dHD}\left(\text{B},\text{ A}\right)\;=\text{max }\left(\text{ b ϵ B}\right)\;[\text{min}\text{ a ϵ A }\left[\;||\text{b}-\text{a}||\;\right] \end{array}$$

### Dosimetric Plan Quality Analysis

Five representative prostate and HN patients were selected from each group, and their AS and MS contours were used to optimize a VMAT treatment plan, respectively, based on institutional planning practice by the same planner using the same beam arrangement. A total of 40 Gy was planned for the prostate patients based on a prospective stereotactic body radiation therapy study (NCT0105913) (22). Conventional simultaneously integrated boost (SIB) with three levels of prescriptions (70, 60, and 54 Gy) was planned for HN cases. For prostate patients, both AS target and OARs were used in the AS plan optimization and evaluation, while clinical target contours and AS OAR contours were used for HN cases. The cumulative dose volume histograms (DVHs), were calculated for AS and MS contours, respectively, based on the dose matrix generated from these two treatment plans. In other words, there are three sets of DVHs generated for comparison. The DVHs calculated using the MS contours based on the treatment plan optimized by the MS contours (labeled as PlanMEvalM) represent the plan quality of the clinical plan. The DVHs calculated using the AS contours based on the plan optimized by the AS contours (labeled as PlanAEvalA) represent the plan dosimetry of using AS contours in the entire planning and evaluation process. The DVHs calculated using the MS contours based on the plan optimized by the AS contours (labeled as PlanAEvalM) were considered as the controlled observation to evaluate the quality of the plan generated by the AS contours.

Dosimetric parameters were derived from each contour's associated DVH curve based on institutional dose constraints, listed in Tables 1, 2, respectively, for prostate and HN. These constraints were used to assist decision-making and quality evaluation for treatment plans. Finally, paired *t*-tests were employed to determine whether statistically significant differences were present between the means of the two groups. A *p* < 0.05 was considered to be statistically significant. In addition to the dosimetric parameters, the plan dose distributions and DVHs were also reviewed by the radiation oncologists and the overall plan quality of the PlanAEvalM were compared with PlanMEvalM and ranked as (1) clinically equivalent or better than the clinical plan, (2) inferior to clinical plan but clinically acceptable, or (3) clinically unacceptable.

**Table 1:** Plan quality evaluation and average achieved values for contours of prostate patients of different dosimetric evaluations (PlanAEvalA, AS optimized plan with AS contour for evaluation; PlanMEvaM, MS optimized plan with MS contour for evaluation; PlanAEvalM, AS optimized plan with MS Contour for evaluation).

**Structure**	**Constraint**	**Achieved value (Mean** **±** **Std) (%)**			**# of plans exceed constraints**		
		**PlanAEvalA**	**PlanMEvalM**	**PlanAEvalM**	**PlanAEvalA**	**PlanMEvalM**	**PlanAEvalM**
CTV	V40Gy ≥ 95%	96.68 ± 4.45	99.51 ± 0.33	99.29 ± 1.30	0	0	0
Bladder	V20Gy ≤ 40%	8.51 ± 5.75	7.15 ± 2.06	7.12 ± 1.66	0	0	0
Bladder	V40Gy ≤ 10%	2.38 ± 2.42	1.58 ± 0.42	2.42 ± 1.48	0	0	0
Femur L	V16Gy ≤ 5%	0.04 ± 0.09	0.25 ± 0.56	0.04 ± 0.09	0	0	0
Femur R	V16Gy ≤ 5%	0.03 ± 0.04	0.25 ± 0.56	0.13 ± 0.19	0	0	0
Rectum	V20Gy ≤ 50%	20.98 ± 5.70	21.34 ± 4.50	20.09 ± 7.05	0	0	0
Rectum	V32Gy ≤ 20%	10.05 ± 5.46	7.38 ± 1.74	6.92 ± 2.21	0	0	0
Rectum	V36Gy ≤ 10%	6.02 ± 3.04	4.82 ± 1.28	4.36 ± 1.56	0	0	0
Rectum	V40Gy ≤ 5%	3.35 ± 1.88	2.03 ± 0.80	1.54 ± 0.95	0	0	0

**Table 2:** Plan quality evaluation and average achieved values for contours of HN patients of different dosimetric evaluations (PlanAEvalA, AS optimized plan with AS contour for evaluation; PlanMEvaM, MS optimized plan with MS contour for evaluation; PlanAEvalM, AS optimized plan with MS Contour for evaluation).

**Structure**	**Constraint**	**Achieved value (Mean** **±** **Std)**			**# of plans exceed constraints**		
		**PlanAEvalA**	**PlanMEvalM**	**PlanAEvalM**	**PlanAEvalA**	**PlanMEvalM**	**PlanAEvalM**
Brain	Max <60Gy	49.41 ± 8.08	57.08 ± 2.50	55.14 ± 2.57	0	0	0
Brainstem	Max <52Gy	29.22 ± 3.58	32.64 ± 2.31	35.44 ± 2.24	0	0	0
Chiasm	Max <52Gy	3.20 ± 0.71	3.81 ± 0.87^*^	3.75 ± 0.48	0	0	0
Cord	Max <45Gy	29.41 ± 1.72	33.54 ± 2.86	34.94 ± 2.34^+^	0	0	0
Esophagus	Mean <25Gy	18.71 ± 7.16	19.03 ± 5.59	22.01 ± 6.44^+^	0	0	1
Larynx	Mean <40Gy	30.58 ± 9.75	29.85 ± 7.18	30.45 ± 7.80	0	0	1
Lips	Mean <20Gy	14.86 ± 3.58	16.56 ± 4.75	18.03 ± 5.94	0	0	2
Mandible	V70Gy <5%	3.01 ± 4.45	2.72 ± 3.46	2.43 ± 3.16	1	1	1
optic nerve L	Max <52Gy	2.76 ± 0.84	3.32 ± 0.92^*^	3.12 ± 0.61	0	0	0
optic nerve R	Max <52Gy	3.14 ± 0.95	3.67 ± 0.96	3.71 ± 0.65	0	0	0
orbit L	Mean <30Gy	2.02 ± 1.00	2.38 ± 1.29	2.32 ± 1.10	0	0	0
orbit R	Mean <30Gy	2.04 ± 0.95	2.38 ± 1.11	2.34 ± 0.97	0	0	0
parotid contralateral	Mean <26Gy	22.80 ± 12.68	24.61 ± 16.62	23.49 ± 12.54	1	1	1
parotid contralateral	V30Gy <50%	26.09 ± 25.89	29.54 ± 32.96	26.24 ± 24.35	1	1	1
Pharynx	Mean <40 Gy	31.40 ± 9.57	42.41 ± 14.59	33.82 ± 9.00	0	3	2
Pharynx	V45Gy <33%	17.04 ± 13.23	40.80 ± 33.48	18.61 ± 20.54	0	3	1

* p < 0.05 between PlanAEvalA and PlanMEvalM pairs.

+ *p < 0.05 between PlanAEvalA and PlanAEvalM pairs*.

## Results

### Geometric Similarity Comparison

**Figure 2:**
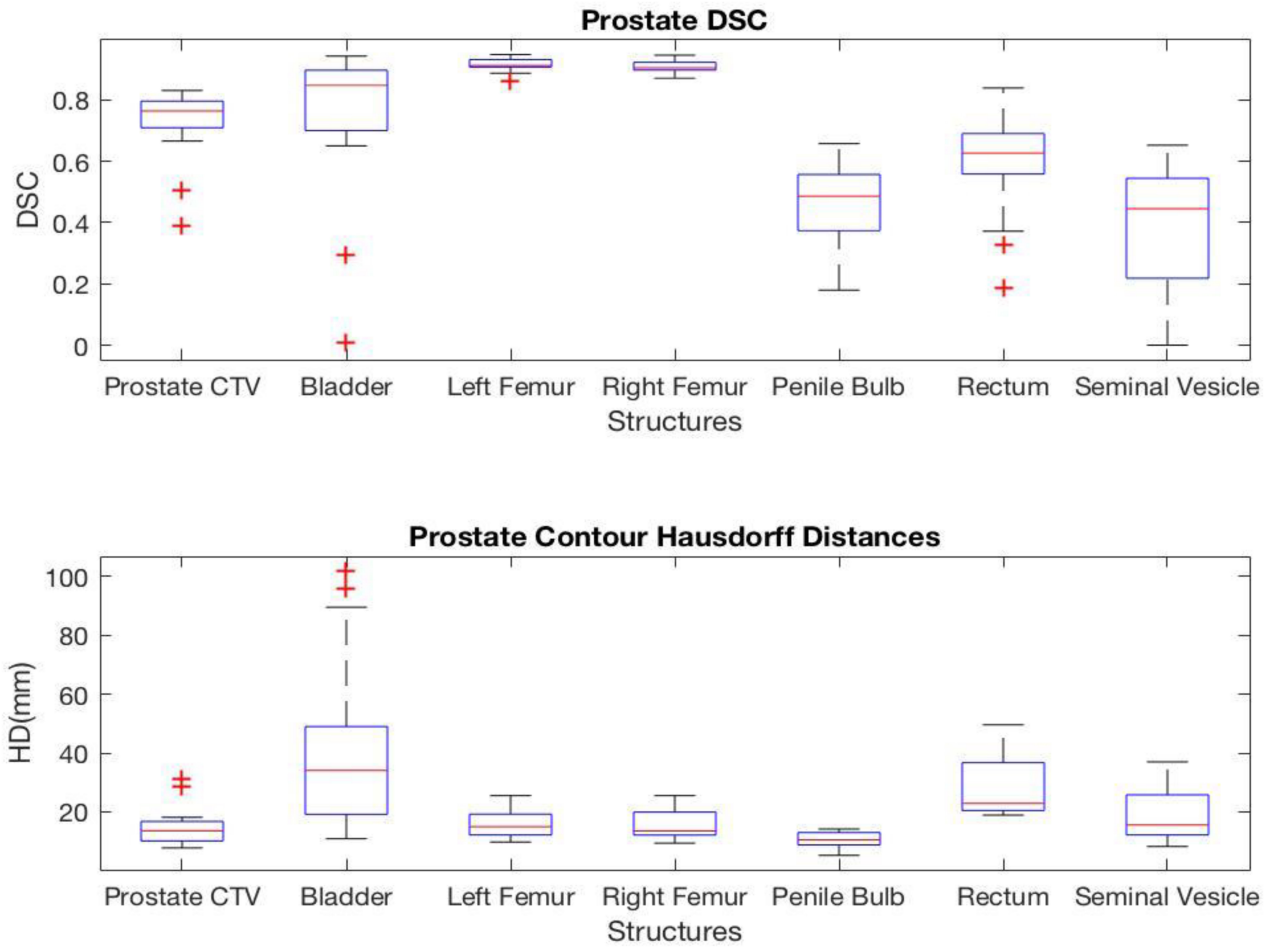
Box plots representing the **(Top)** DSC and **(Bottom)** HD for each prostate structures.

For prostate structures, the highest correlation was found in the femoral heads with average DSC values of 0.92 and 0.91 and average HD values of 15.6 and 15.7 mm for left and right femoral heads, respectively. However, the seminal vesicle and penile bulb showed much lower similarity with average DSC of 0.38 and 0.47, and average HD of 18.9 and 10.4 mm, respectively. A complete list of average values measuring the geometric correlation between the AS and MS contours are shown in Figure 2.

**Figure 3:**
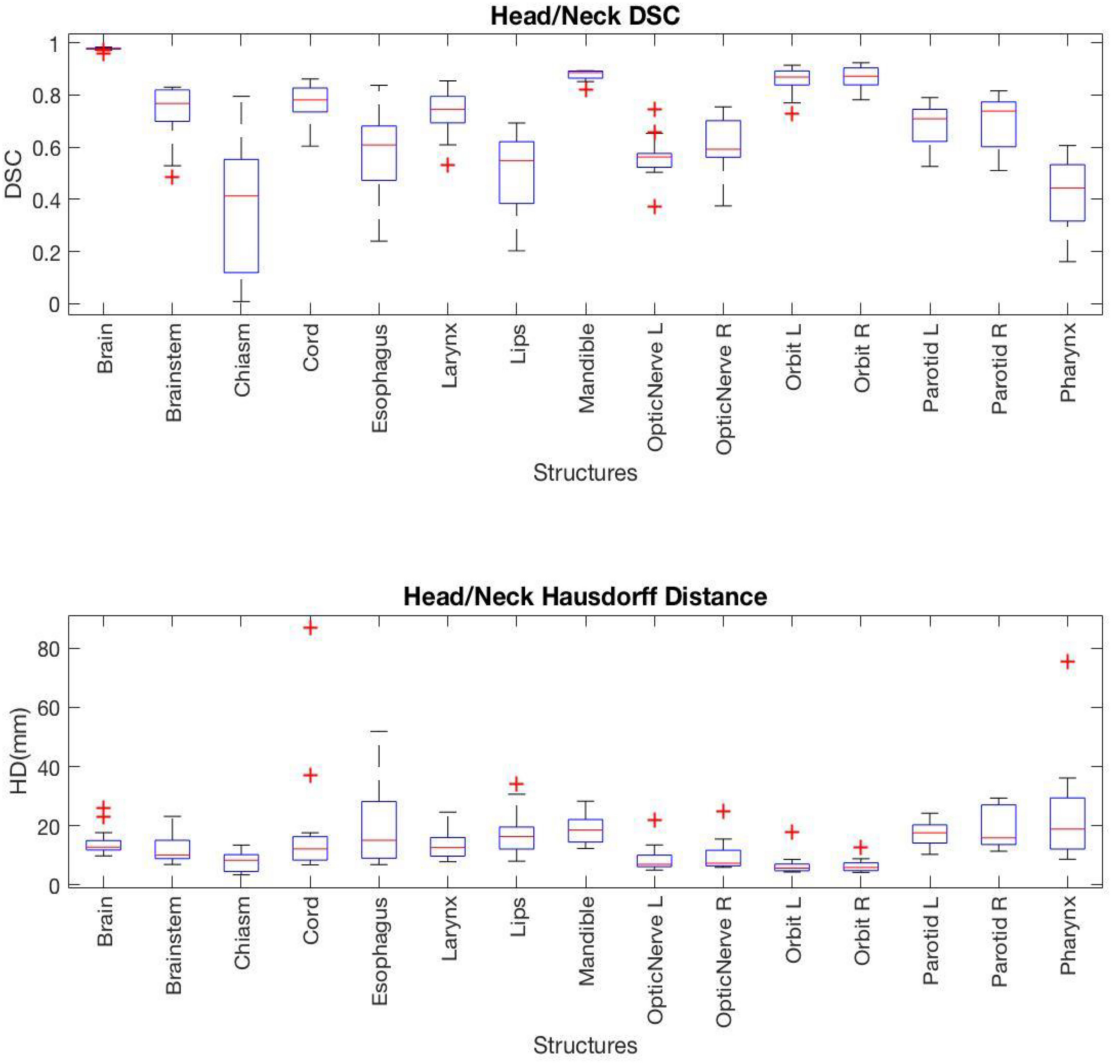
Box plots representing the **(Top)** DSC and **(Bottom)** HD for each HN structure.

For HN cases, several structures including brain, mandible, left orbit, and right orbit showed a high level of correlation with average DSC of 0.98, 0.88, 0.86, and 0.87, and average HD of 14.4, 18.8, 6.8, and 6.5 mm, respectively. A few of the HN structures had lower geometric correlations between the AS and MS contours. For example, chiasm, lips, and pharynx had average DSC values of 0.36, 0.51, and 0.43, and average HD values of 8.0, 17.6, and 23.7 mm. Figure 3 provides a complete list of DSC and HD values for HN contours.

The DSC and HD values vary by structures with a noticeably lower geometric correlation in small structures such as chiasm, optical nerves, and elongated structures such as pharynx and esophagus. It is also important to note that HD measures the worst match across all boundary pairings, and therefore is highly sensitive to small regions and affected by the size as well as the shape of the structure under evaluation.

### Plan Quality Comparison Results

**Figure 4:**
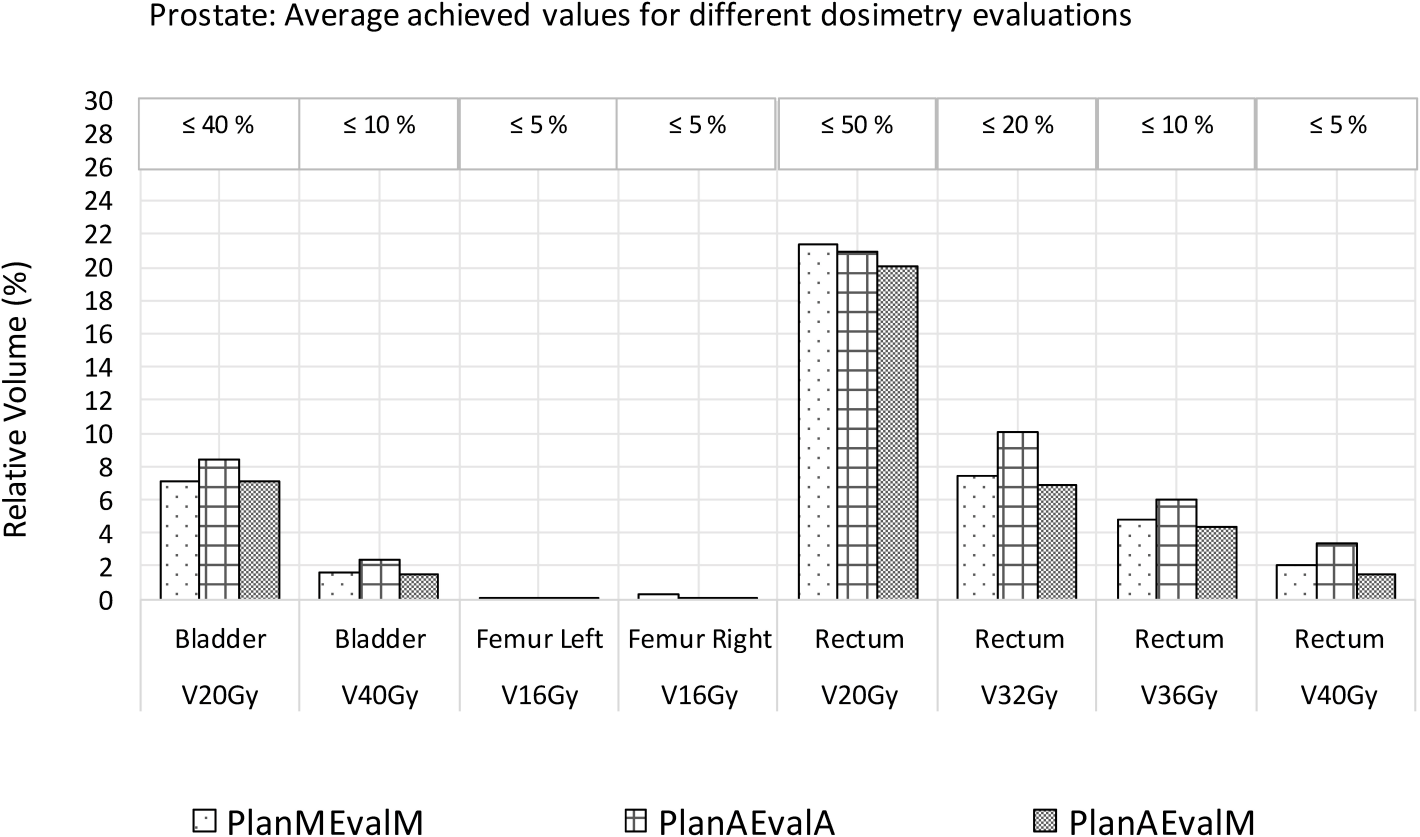
Comparison of dosimetric plan quality values of AS and MS contours for prostate. (PlanAEvalA, AS optimized plan with AS contour for evaluation; PlanMEvaM, MS optimized plan with MS contour for evaluation; PlanAEvalM, AS optimized plan with MS Contour for evaluation).

**Figure 5:**
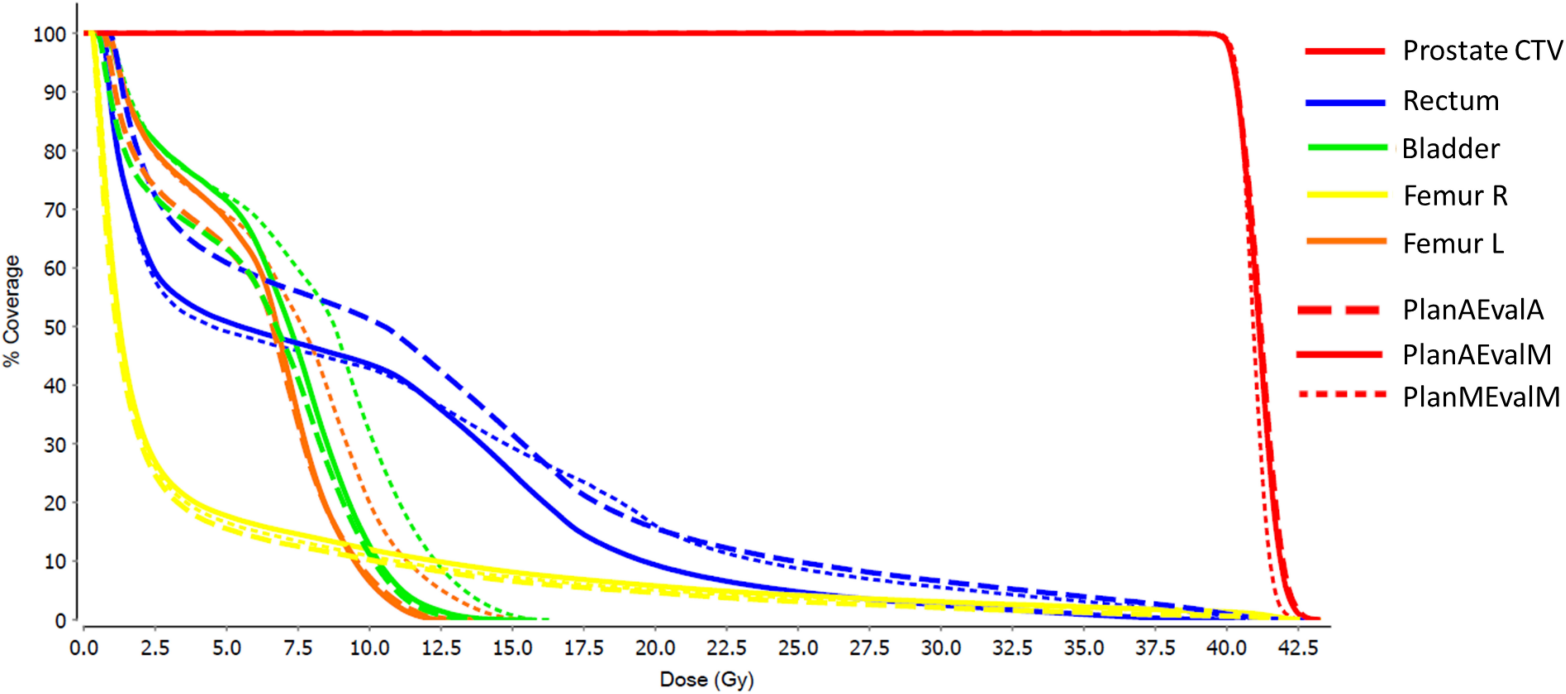
DVHs of a representative prostate patient with three different plans (PlanAEvalA, AS optimized plan with AS contour for evaluation; PlanMEvaM, MS optimized plan with MS contour for evaluation; PlanAEvalM, AS optimized plan with MS Contour for evaluation).

For prostate patients, the CTV V100 ≥ 95% had a difference of 2.8% between PlanAEvalA and PlanMEvalM plans, while the corresponding difference between PlanAEvalA and PlanAEvalM is 2.6%. All the normal tissue dosimetric constraint differences were <2% between PlanAEvalA and PlanMEvalM pairs, as well as PlanAEvalA verse PlanAEvalM pairs, except for the constraint of Rectum V32Gy <20% of which the difference is 2.7% between PlanAEvalA and PlanMEvalM and 3.2% for the PlanAEvalA vs. PlanAEvalM pair, respectively. The average achieved dosimetric values for all the OARs of prostate patients are compared in Figure 4. The acceptable constraint value is also listed on the top of each constraint parameter in the figure. None of these plans had any OAR dosimetry exceeding the acceptable constraints, as shown in Table 1. The paired *t*-tests failed to identify a significant difference between the achieved dosimetric parameters between these plan pairs, suggesting that the AS generated plans achieve similar performance as the MS contour in dose optimization and evaluation process for prostate planning. DVHs of a representative prostate case is shown in Figure 5. For this particular case, the DVHs of CTV and bladder generated from three plans (PlanAEvalA, PlanMEvalM and PlanAEvalM) agreed very well, even with a moderate DSC of 0.83 and 0.87, respectively. Although both femoral heads had relatively higher DSC > 0.90, noticeable DVH differences were evident between PlanAEvalA, and PlanMEvalM plans below the dose constraint (16 Gy) region, probably because only one dose constraint (V16Gy <5%) was used in dose optimization. The rectum DSC of this patient was 0.72. Nevertheless, the DVHs from PlanAEvalA and PlanMEvalM agree very well in the high dose region corresponding to the multiple-dose constraints used in the optimization. The DVH from PlanAEvalM indicated that the dosimetry generated from the AS contour spared the actual rectum more aggressively. The overall plan quality was reviewed by radiation oncologists with comprehensive evaluation of dose distribution and DVHs in addition to dosimetric parameters. The assessment indicated that all the plans optimized from AS contours were clinically equivalent or better than its counterpart clinical plan.

**Figure 6:**
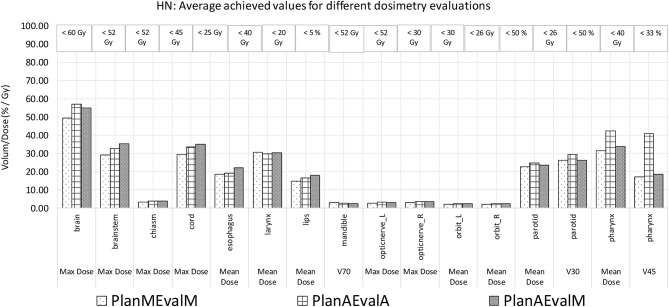
Comparison of dosimetric plan quality values of AS and MS contours for HN patients. (PlanAEvalA, AS optimized plan with AS contour for evaluation; PlanMEvaM, MS optimized plan with MS contour for evaluation; PlanAEvalM, AS optimized plan with MS Contour for evaluation).

**Figure 7:**
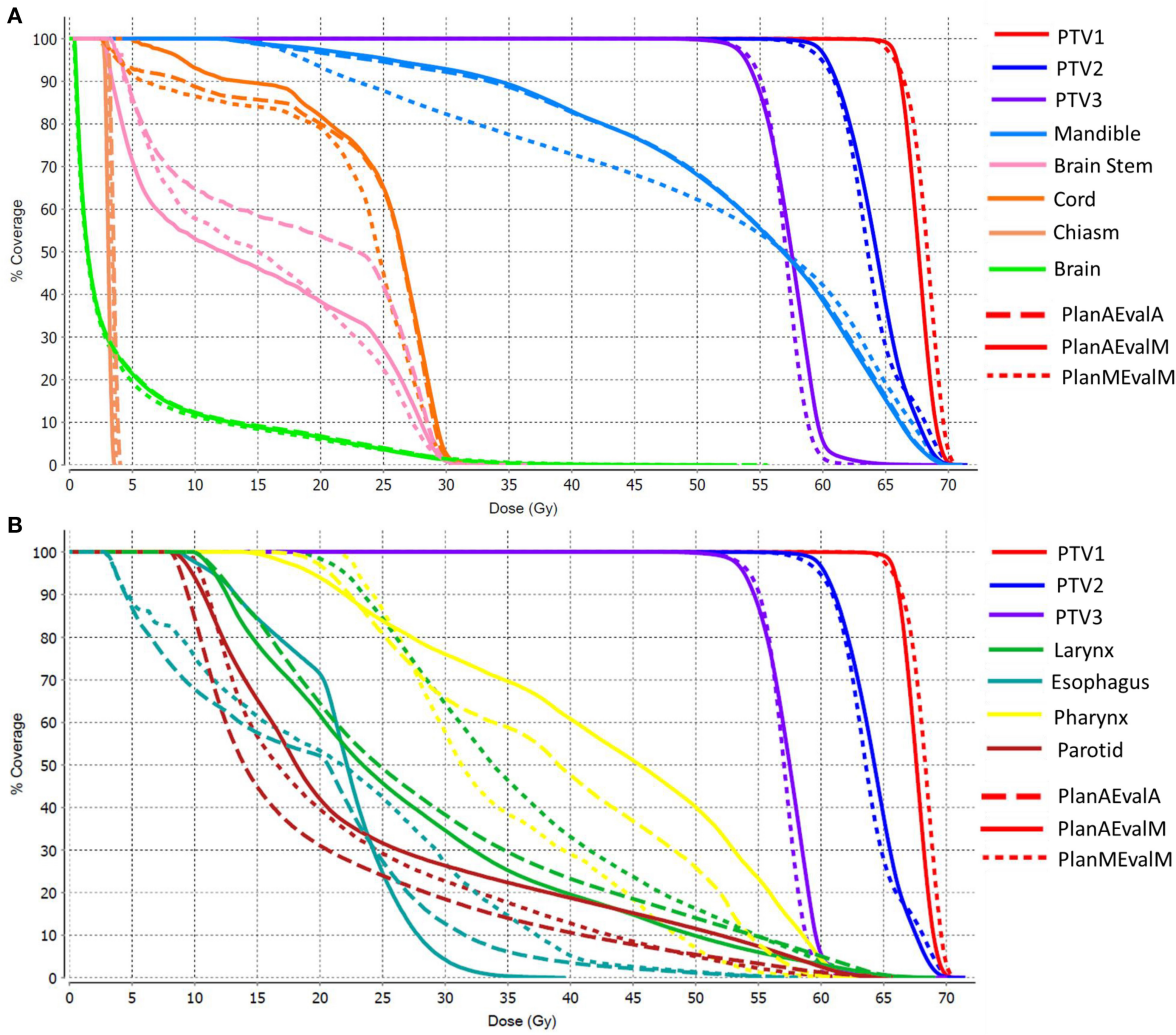
DVHs of a representative HN patient **(A)** DVHs of structures where maximum point dose is the primary dose constraint **(B)** DHVs of structures where mean dose is of primary interest as dose constraint. (PlanAEvalA, AS optimized plan with AS contour for evaluation; PlanMEvaM, MS optimized plan with MS contour for evaluation; PlanAEvalM, AS optimized plan with MS Contour for evaluation).

The average achieved dosimetric values of the five HN patients are listed in Table 2 and compared in Figure 6. The largest dissimilarity was seen in the pharynx mean dose <40 Gy and pharynx V45 <33%, constraints with differences of 11 Gy, and 23.75% between the PlanAEvalA and PlanMEvalM pairs, respectively. The differences were relatively smaller (8.59 Gy and 22.19%) between the PlanAEvalA and PlanAEvalM plan pairs. The differences in mean dose values between the PlanAEvalA and PlanMEvalM plans were statistically significant for two constraints, including chiasm maximum dose (*p* = 0.04) and left optical nerve maximum dose (*p* = 0.02), although the absolute mean dose different was only 0.61 and 0.57 Gy, respectively. Statistically significant differences between PlanAEvalA and PlanAEvalM plans were found for cord maximum dose (*p* = 0.01) and esophagus mean dose (*p* = 0.04) with absolute dose differences of 1.4 and 2.98 Gy, respectively. The numbers of patients of which the achieved OAR dose exceeding the acceptable tolerance are listed in Table 2. The pharynx was the OAR with the most patients exceeding the tolerance in PlanAEvalA plans compared with their corresponding clinical PlanMEvalM plans. For the esophagus, larynx, and lips, none of the PlanAEvalA plans had dose exceeding the acceptable constraint level. However, one or two of these plans became unacceptable if the same plan is evaluated using the MS contours. DVHs from a representative HN case is shown in Figure 7. For structures that only maximum dose constraints were considered, such as the brain, cord, and brainstem, the DVHs agreed well at the maximum dose region of each structure, although noticeable dose difference can be seen in the lower dose range as shown in Figure 7A. For other structures where mean doses are of interest such as larynx, esophagus, and pharynx, the DVHs exhibited wide variations throughout the entire dose range as demonstrated in Figure 7B. The comprehensive plan quality evaluation by radiation oncologists revealed that four of the five HN plans optimized from AS contour were considered as clinically equivalent or better than its counterpart clinical plan. One plan was deemed as inferior to clinical plan but clinically acceptable.

## Discussions

As automatic segmentation tools become a more appealing and plausible alternative to manual segmentation by experts, it is crucial that these applications undergo a detailed and comprehensive evaluation. Most reported evaluation metrics are based on geometric features, including moment, overlap and distance-related parameters, such as the DSC and HD, as used in this study. A major issue of these geometric metrics is that they are not directly related to the treatment plan dosimetry, making it challenging to evaluate the accuracy and effectiveness of using automatically segmented contours in dose optimization and plan evaluation.

In this study, atlas-based segmentation of a commercially available software was evaluated comparatively to MS contours for both geometric correlation and dosimetric plan quality performance. It was found that the target and OARs of the prostate patients were segmented with moderate to high geometric accuracy, and DSC of most structures was in the range of 0.6–0.92. Several structures, including the brain, mandible, left orbit, and right orbit, showed a high level of geometric correlation for the HN cases. Substantial variations of geometric correlations were observed for different structures in this study, which is consistent with the literature (2, 7, 8, 10, 23). A noticeable trend of inferior geometric correlation was identified for relatively small structures such as chiasm, optical nerves or elongated structures such as pharynx and esophagus. The inconsistency in relatively small organs for HN subjects may be attributed to the specific atlas selection method where global intensity similarity is used as the matching metric and, consequently, the contributions of relatively small local regions are discounted. This issue may be alleviated by utilizing methods such as a multi-atlas subject selection method or a multiple-step atlas selection technique (24). The geometric discrepancy between AS and MS contours may have been caused by the inconsistencies in MS contours. For example, large variations in the superior-inferior ranges of cord and femoral neck MS contours occurred between patients. This became problematic when the selected atlas subject's contour varied largely in length compared to the associated MS contour.

In theory the accuracy of structure contours directly impacts the plan optimization and calculation of DVH, and thus the treatment plan quality evaluation and decision-making process. This study was primarily motivated by the fact that there is a scarcity of studies performing any type of dosimetric evaluation of AS contours. Therefore, the main focus of this research was to further determine whether the contours created by AS could produce comparable dosimetric results to MS contours when analyzed with respect to dosimetric plan quality metrics. It was found that the plan dosimetry optimized based on AS contours were in reasonable agreement with the plan generated from MS contours for prostate contours, even though the geometric accuracy were in the moderate range for several structures such as bladder and rectum, suggesting that geometric metrics may not be directly used to infer with dosimetry performance. The absence of a statistically significant difference between AS and MS contours for the large majority of dosimetric values demonstrated the potential of AS contours in dose optimization and evaluation of treatment plan quality. However, a select few of the dosimetric values did show large enough differences for HN cases, especially for small elongated structures such as pharynx and esophagus, which also had relatively low DSC values. On the other hand, the dosimetric differences of organs with low geometric accuracies such as chiasm and optical nerves are relatively small between AS and MS plans because they are located distant to the target and high dose region. Again, this demonstrates the discrepancy between geometric metrics and dosimetric performance and emphasizes the importance of developing dosimetric related evaluation metrics.

The discrepancy between geometric and dosimetric performance reflects the complex interplay between structure geometry and dose distribution. The dosimetry performance not only depends on geometric accuracy, but is also heavily impacted by spatial dose distribution and gradient. For a structure that is far away from the high dose region, even if there is notable difference in the dosimetric metrics between the AS and MS contours, their absolute dosimetric values can be too low to impact the plan evaluation and decision making. This was evident in the organs such as chiasm and optical nerves for the HN site in this study. In addition, if an organ is located in a high dose region with low dose gradient, its dosimetric metrics may have high absolute value, but minor variation related to geometric shape change. Furthermore, each dosimetric parameter (i.e., maximum, mean, or volume based parameter) has different dependency and sensitivity to geometric variation depending on if it extracts point or volume based dosimetry. For example, for a structure located at a high dose gradient region, the maximum dose may vary more than the mean dose when the size structure changes. Overall the complex interplay between structure geometry and dose distribution emphasizes the importance of developing evaluation metrics integrating both geometry and dosimetry assessment.

The clinical feasibility and validity of the AS-driven approach depends on the treatment site, the segmentation of structure for the site, and the stringency of the clinical criteria, as shown by the differences between the prostate and HN sites. The varying performance of AS contours among structure sets suggests a different approach (i.e., applying automatic segmentation to generate a subset of contours where AS performs consistently well, and reserving the clinical effort to the complement subset that may be more sensitive and subject to larger error or variation).

There are some limitations with this study. First, atlas-based segmentation was evaluated in this study, which may not reflect the most state-of-the-art performance of the automatic segmentation, particularly with sophisticated shape modeling or deep learning methods. However, since the primary goal of this study was not to assess the geometric accuracy, we chose the atlas-based segmentation which is widely available in most commercial products. With a continuous translation of advanced segmentation methods to clinical practice, it is reasonable to expect both geometric and dosimetric performance to be improved further. Second, the dosimetric evaluation was performed with a subset of the patients in this study. Although it clearly demonstrated the discrepancy between geometric metrics and dosimetry performance, a larger pool of patient samples in future studies would be beneficial to characterize the dosimetry performance of each individual structure.

## Conclusion

Variations of AS performance among structures alludes to a differential approach of using AS on a structure subset and focusing MS on the rest. The discrepancy between geometric and dosimetric-end-point driven evaluation also indicates the potential utility of AS contours in predicting plan quality, albeit geometrically imprecise.

## Data Availability Statement

The datasets presented in this article are not readily available publicly in compliance with institutional policy. Requests to access the dataset should be directed to the corresponding author.

## Ethics Statement

The studies involving human participants were reviewed and approved by UCLA IRB Review Committee. Written informed consent for participation was not required for this study in accordance with the national legislation and the institutional requirements.

## Author Contributions

MC, BS, VY, KS, AK, RC, YY, and DR contributed to authoring the manuscript. MC, BS, VY, AK, RC, and DR contributed to data collection and analysis. All authors contributed to the article and approved the submitted version.

## Conflict of Interest

The authors declare that the research was conducted in the absence of any commercial or financial relationships that could be construed as a potential conflict of interest.
